# Action classification for endoscopic pituitary adenoma resection: a consensus-based study

**DOI:** 10.1007/s11548-026-03614-2

**Published:** 2026-04-11

**Authors:** Joachim Starup-Hansen, Danyal Z. Khan, Adrito Das, Joao Paulo Almeida, Sophia Bano, Anouk Borg, Kevin Cleary, Neil Dorward, Juan C. Fernandez-Miranda, Eduardo Torres-Rodríguez, Danail Stoyanov, Recai Yilmaz, Peter Weir, Daniel A. Donoho, Hani J. Marcus

**Affiliations:** 1 Victor Horsley Department of Neurosurgery, National Hospital for Neurology and Neurosurgery, Queen Square, London, UK; 2 UCL Hawkes Institute, University College London, London, UK; 3 Department of Neurosurgery, Indiana University, Indianapolis, IN USA; 4 Sheikh Zayed Institute for Pediatric Surgical Innovation, Children’s National Hospital, Washington, DC 20010 USA; 5 Department of Neurosurgery, Stanford University, Palo Alto, CA USA; 6 Division of Pediatric Neurosurgery, Center for Neuroscience Research, Children’s National Hospital, Washington, DC USA; 7 Department of Neurosurgery, John Radcliffe Hospital, Oxford, UK

**Keywords:** Artificial intelligence, Endoscopic neurosurgery, Pituitary surgery, Surgical skill assessment, Surgical video annotation, Operative workflow

## Abstract

**Purpose:**

Pituitary adenoma resection via the endoscopic transsphenoidal approach is technically demanding, with outcomes influenced by surgical skill. However, the association between technique and outcomes remains poorly defined. Existing workflow analyses focus on broad procedural steps and phases, but a more detailed, action-level approach is needed to capture skill-related variation. While AI shows promise in automating workflow analysis, its use at the action level is limited. This study develops and validates a reproducible action-level classification ontology for endoscopic pituitary adenoma resection, establishing the structured annotation foundation required for future AI-based workflow and skill analysis.

**Methods:**

Endoscopic videos of primary pituitary adenoma resections were collected from two high-volume international pituitary centres. A multi-disciplinary panel of neurosurgeons and data scientists iteratively reviewed and annotated surgical actions to establish a standardized classification system. Actions were categorized into triplets (instrument, target, verb), with additional temporal annotations. To evaluate framework reliability, an independent annotator followed a structured annotation guide, and inter-annotator agreement was measured using Cohen’s Kappa.

**Results:**

A consensus-based classification ontology was developed, comprising 9 verbs, 12 instruments, and 7 targets from the review of 18 endoscopic pituitary adenoma resections (9 microadenomas, 9 macroadenomas). Action distribution differed between micro- and macroadenomas, with grasping being the predominant action in microadenomas (72% of right-hand frames) and blunt dissection and traction dominating macroadenomas. The left hand primarily performed non-meaningful movements (88% of macroadenoma frames, 55% of microadenoma frames), while the right hand was responsible for more deliberate tool–tissue interactions. Inter-rater reliability analysis demonstrated substantial to near-perfect agreement (*κ* = 0.69–0.95), confirming the reproducibility of the annotation system.

**Conclusion:**

While acknowledging that conclusions remain limited by dataset size and validation stability, this study establishes a robust and interpretable action classification ontology for pituitary adenoma resection. The ontology enables high-quality, standardized labelling for future computer-vision AI works, and lays the groundwork for evaluating whether action-level annotation improves surgical outcome prediction and automated skill assessment.

## Introduction

Pituitary adenomas are common brain tumours that significantly impact patient morbidity, mortality, and quality of life [1–4]. Endoscopic transsphenoidal surgery remains the standard of care for resection; however, surgical outcomes vary considerably and are closely linked to the surgeon’s technical performance [1–4]. Despite this association, the precise technical determinants of surgical performance and their relationship to clinical outcomes remain poorly defined. Artificial intelligence (AI) has been proposed as a tool to identify these determinants through the automated analysis of surgical video data [5–9]. Yet, the development of AI-driven surgical video analysis first requires a robust understanding of an operation’s workflow. Operative workflow analysis describes the deconstruction of an operation into its fundamental building blocks, including its distinct phases, steps, tasks, and actions [10]. Phases are major events occurring during a surgical procedure, composed of several steps [11]. Steps (e.g. *tumour resection*) may be composed of tasks (e.g. *removing tumour tissue*) executed through specific actions. Actions are defined as the interaction of instruments (e.g. *pituitary rongeur*) with a target (e.g. *tumour*) through verbs (e.g. *grasping*) [12–14]. The application of AI to this hierarchical framework may enable objective, scalable, and automated surgical performance assessment, with potential implications for both surgical education and patient outcomes.

Our group has previously developed an international consensus framework, detailing key surgical phases, steps, instruments, and anatomy for endoscopic pituitary adenoma surgery [15]. This has enabled the training of AI models capable of accurately recognizing the phases and steps of a pituitary adenoma resection [6, 7]. Yet, recent work interrogating the relationship between step-specific metrics and clinical outcomes found few significant correlations [16]. Instead, a broader analysis of step-specific surgical skills (assessed manually by humans) identified meaningful patterns in outcomes, as higher surgical skills in the tumour resection and closure steps were correlated with improved visual outcomes and CSF leak rates, respectively [16]. This suggests that while step-specific quantitative metrics are not enough to predict operative outcomes, a more detailed analysis of surgical movements may offer a solution.

Expanding on these works, we hypothesize that AI models capable of analysing the sub-components of the tumour resection step of the endoscopic transsphenoidal approach (i.e. actions) would identify the differences between operations which account for these variations in operative outcomes. Beyond outcome prediction, such action analysis may assist in surgical skill training via the development of quantitative performance metrics [17] and lay foundations for even more granular analyses in the future (e.g. Sergemes or gestures) [10]. Examples of the development of action analysis framework can be found in other surgical domains including Urology and General surgery, but not in neurosurgery [9, 18, 19].

Adapting these works to a novel domain, the present work describes the development of a universal classification ontology [20] for the actions of the endoscopic transsphenoidal approach to a pituitary adenoma resection. Acknowledging consensus statement recommendations for universal frameworks, we generate this ontology through an international, multi-disciplinary, multi-centric, consensus-based approach, and provide annotation guides to facilitate standardized data curation and AI model development [5]. The aim of this study is to develop a reproducible and consensus-based action-level ontology for pituitary surgery, as a prerequisite for downstream AI applications. Rather than reporting model performance, this work defines the structured labelling framework that will allow future studies to train and benchmark action-recognition models at scale.

## Methods

### Cases included

Endoscopic videos of primary pituitary adenoma resections performed via the endoscopic transsphenoidal approach were retrieved from two prospectively maintained operative video databases from two international pituitary centres. The tumour resection step was selected for analysis due to its close correlation with patient outcomes in prior works and considered as per the pituitary society’s operative workflow consensus [15]. Cases without complete footage of the tumour resection step were excluded from the analysis. For the video included in the analysis ethical approval was granted by the Institutional Review Board (IRB) at University College London (17819/011) with informed participation consent. Cases were performed by an attending surgeon with trainee assistance.

### Development of action classification system

The action framework was generated through the review of operative videos combined with rounds of multi-disciplinary consensus discussion. A steering group (*n* = 13) was established of expert pituitary 6 surgeons from four institutions (USA *n* = 3, Europe *n* = 1), three neurosurgery residents with video-annotation experience in pituitary surgery from two institutions (USA *n* = 1, Europe *n* = 1), and three data scientists from two institutions (Europe *n* = 1, USA *n* = 1).

To generate the preliminary task and action classification framework, two independent annotators manually reviewed and segmented the tumour resection step of six operative videos (3 microadenoma resection, 3 macroadenoma resections), conducted at a single UK institution (referred to previously as Europe *n* = 1). All videos were uploaded using Touch Surgery™ Ecosystem, an AI-powered surgical video management and analytics platform provided by Medtronic [21]. Data annotation was performed using Encord™ [22]. Both preliminary annotators were neurosurgery residents, with practical experience in pituitary surgery [19]. Actions were defined using the framework of Padoy et al. [9] and thus split into “triplets” of: *instruments* (e.g. ring curette), interacting with a *target* (e.g. tumour), through a *verb* (e.g. *blunt dissection*). Additional unique temporal annotations, including *instrument not in frame* and *ineffective movement*, were included to account for video segments in which an instrument was out-of-frame or not seemingly interacting with a tissue target through purposeful movements, respectively.

Next, the preliminary framework was scrutinized by the steering group for its clinical applicability and machine readability, iterated through multiple rounds of review via e-mail and consensus meetings. Following consensus, six unseen videos from the US institution (three microadenomas and three macroadenomas) were manually segmented using the updated framework. If a new action or task was identified which had yet to be classified in the previous framework, this was proposed as a suggested modification to the expert panel. This process was repeated until saturation was reached, defined as the annotation of a new batch of unseen videos, resulting in no further additions to the framework.

### User-guide and inter-annotator consistency scoring

Considering the importance of labelling instructions on the quality of data in biomedical image analysis [23, 24], a video-based user-annotation guide was developed to outline the expected annotation workflow and the agreed appearance of important actions and tasks. To evaluate the interpretability of the framework, the user guide was provided to an annotator with limited experience in endoscopic pituitary video annotation, who then annotated a common dataset. An inter-annotator reliability assessment was conducted followed by a review of discrepancies, resulting in either the update of the annotation guide, or the ontology framework. Inter-rater reliability was assessed using Cohen’s Kappa (*κ*), which quantifies agreement beyond chance. *κ* was calculated for verbs, targets, instruments, and triplets, separately for left- and right-hand annotations. Agreement was classified according to the Landis and Koch scale: poor (< 0.20), fair (0.21–0.40), moderate (0.41–0.60), substantial (0.61–0.80), and almost perfect (> 0.81) agreement [25]. To quantify variability, 95% confidence intervals (CIs) for *κ* were computed. All analyses were conducted in R (v 2022.07.2, R Foundation for Statistical Computing, Vienna, Austria). *κ* values were reported separately for left- and right-hand annotations across surgical videos to assess laterality effects in annotation reliability.

### Data analysis

Following dataset consensus, four endoscopic videos (two micro- and two macroadenomas) were annotated for their tumour resection step by an experienced annotator. The annotated triplets, verbs, targets, and instruments were analysed for their incidence and proportional frame composition. Left- and right-hand triplets were considered separately, as were micro and macroadenomas. Due to the low video number, no comparative statistical analysis between hands or instruments was conducted.

## Results

### General

The final framework was developed through four rounds of discussion and the review of 18 primary endoscopic transsphenoidal resections of pituitary adenomas (9 microadenomas, 9 macroadenomas). The panel included representation from one UK and one US high-volume pituitary centre. The final action ontology, agreed upon by 100% of panel members in the final round of discussion included 9 verbs, 12 instruments, and 7 targets. Table 1 depicts the action classification framework.

**Table 1:** Action classification framework for the tumour resection step of a pituitary adenoma resection

Verb	Instrument	Target
Blunt dissection Sharp dissection Traction Grasping Suction Visualization Application Coagulation Irrigation *Non-meaningful movement	Suction tip Ring curette Dissector Blunt hook Pituitary rongeur Patty Cupped forceps Fluid haemostats Bipolar Saline syringe Knife Scissors	Tumour Dura Gland Arachnoid Sellar cavity Clival recess Extra-clival sphenoid sinus

*Instrument is in frame, but is not performing a meaningful tool–tissue interaction

**Fig. 1:**
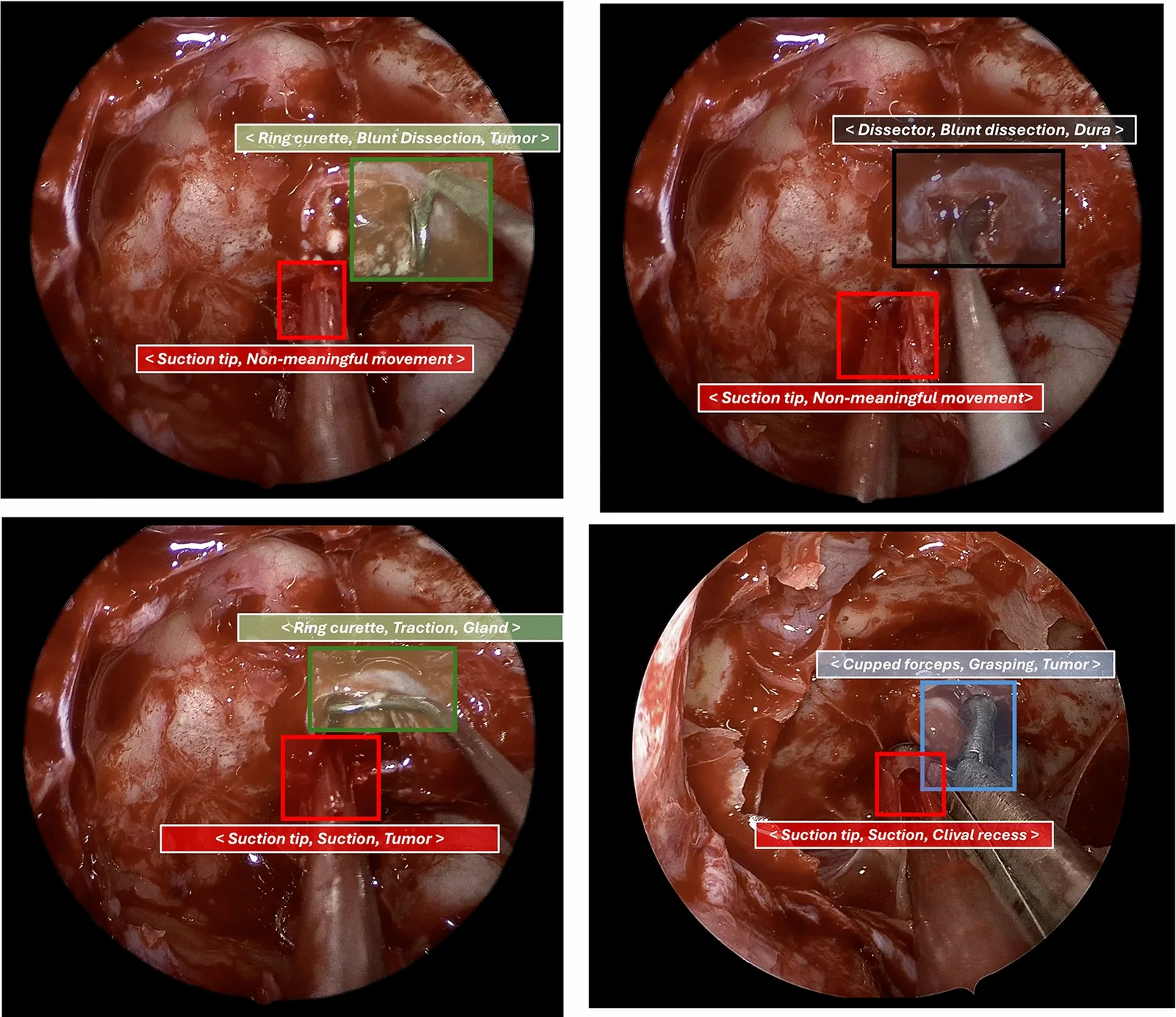
Depicting four sample annotations of left- and right-hand action triplets across the pituitary adenoma resection step

The key annotation events for operative videos included: (i) the instrument entering into frame, (ii) the instrument performing a meaningful tool–tissue interaction (target and verb assigned), (iii) any component of the action triplet changing, and (iv) the instrument moving out-of-frame. To maintain consistency, the “left” action triplet was assigned to the suction tip, whereas the “right” action triplet was assigned to the other instrument. In situations where neither instrument was a suction tip, the instrument which replaced the suction (assuming the right triplet remained in frame) was assigned as the left triplet. Figure 1 demonstrates four sample action-triplet frames at different stages of the tumour resection step.

### User-guide and inter-annotator consistency

Once a final ontology was established, a video-based user-annotation guideline was developed. The guideline included videos covering the expected annotation workflow and common triplets. The user guide was made available to two independent annotators with observational experience in endoscopic pituitary surgery, who labelled a dataset of four videos in duplicate. As shown in Table 2, mean kappa scores demonstrated substantial agreement for 5/8 classes and near-perfect agreement in 3/8 classes.

**Table 2:** Depicting inter-rater agreements for an annotated dataset of four pituitary adenoma resections. CI = confidence interval

Class	Mean Kappa	Mean Kappa 95 CI	Mean Kappa 05 CI
Anatomy: left hand	71.8	71.4	72.3
Anatomy: right hand	80.1	79.7	80.4
Instrument: left hand	95.6	95.5	95.8
Instrument: right hand	94.5	94.3	94.7
Verb: left hand	82.8	82.5	83.2
Verb: right hand	70.4	70	70.8
Triplet: left hand	69.7	69.4	70.1
Triplet: right hand	75.8	75.5	76.1

### Triplet distribution

The final annotated dataset can be found at https://doi.org/10.5522/04/28664990. Across the tumour resection step of the four videos analysed with the final ontology, 281 discrete annotations were produced, of which 178 were considered meaningful triplets. The triplet distribution across frames in the final dataset is listed in Table 3. Tables 4 and 5 list the triplet distributions between micro- and macroadenomas.

**Table 3:** Triplet annotations and their count appearance across the frames finally analysed dataset

Instrument		Verb		Target	
Name	Count (%)	Name	Count (%)	Name	Count (%)
Suction tip	78,978 (71)	non-meaningful movement	43,681 (66)	Tumour	30,786 (78)
Ring curette	17,401 (16)	Blunt dissection	6459 (10)	Dura	4368 (11)
Cupped forceps	4737 (4)	Grasping	5431 (8)	Clival recess	1726 (4)
Spatula dissector	3692 (3)	Traction	4300 (7)	Extra-clival sphenoid sinus	1254 (3)
Pituitary rongeur	2697 (2)	Irrigation	3878 (6)	Gland	1195 (3)
Saline syringe	1536 (1)	Sharp dissection	880 (1)	Arachnoid	283 (1)
Scissors	1302 (1)	Application	592 (1)		
Liquid haemostat	968 (1)	Coagulation	469 (1)		
Blunt hook	26 (< 1)

**Table 4:** Incidence and frame duration of triplets across annotated macroadenoma tumour resections, split according to hand of use

Right hand			Left hand		
Name	Frames (%)	Incidence (%)	Name	Frames (%)	Incidence (%)
Ring curette, blunt dissection, tumour	22.9	42.1	Suction tip, suction, clival recess	34.5	23.3
Ring curette, blunt dissection, gland	18.4	1.3	Suction tip, suction, tumour	26.3	40
Ring curette, traction, tumour	12.8	17.1	Suction tip, traction, dura	20.7	3.3
Spatula dissector, blunt dissection, dura	11.5	3.9	Ring curette, blunt dissection, tumour	6.8	10
Saline syringe, irrigation, tumour	9.2	5.3	Suction tip, traction, tumour	2.7	3.3
Fluid haemostat, application, tumour	6.7	2.6	Suction tip, suction, gland	2.5	6.7
Cupped forceps, grasping, tumour	5.3	1.3	Saline syringe, irrigation, tumour	2.3	3.3
Pituitary rongeur, grasping, tumour	5.2	15.8	Spatula dissector, traction, dura	2.1	3.3
Scissors, sharp dissection, dura	3.8	1.3	Suction tip, blunt dissection, tumour	2	3.3
Ring curette, traction, gland	1.8	6.6	Spatula dissector, blunt dissection, dura	0.1	3.3
Saline syringe, application, tumour	1.4	1.3			
Ring curette, traction, dura	0.9	1.3			

Incidence reflects the proportional frequency of triplet appearance. Frames represent the proportion of frames in which a given triplet was present, averaged across all analysed videos

**Table 5:** Incidence and frame duration of triplets across annotated microadenoma tumour resections, split according to hand of use

Right hand			Left Hand		
Name	Frames (%)	Incidence (%)	Name	Frames (%)	Incidence (%)
Cupped forceps, grasping, tumour	48.5	17.6	Suction tip, suction, tumour	38.9	26.3
Spatula dissector, blunt dissection, dura	13.3	8.8	Suction tip, traction, tumour	25.2	5.3
Pituitary rongeur, grasping, tumour	9.9	2.9	Suction tip, suction, sellar cavity	7.4	2.6
Scissors, sharp dissection, dura	8.1	2.9	Suction tip, suction, gland	6.8	13.2
Spatula dissector, traction, dura	4.2	14.7	Suction tip, sharp dissection, arachnoid	4.9	2.6
Ring curette, blunt dissection, tumour	3.8	17.6	Suction tip, suction, clival recess	4.4	5.3
Spatula dissector, traction, gland	3.8	5.9	Suction tip, traction, dura	3.3	2.6
Saline syringe, irrigation, gland	3.5	2.9	Spatula dissector, blunt dissection, dura	2.8	2.6
Cupped forceps, traction, gland	1.8	5.9	Bipolar, application, clival recess	2.3	2.6
Ring curette, traction, gland	1.6	17.6	Bipolar, coagulation, tumour	1.9	2.6
Spatula dissector, sharp dissection, gland	1.4	2.9	Pituitary rongeur, grasping, tumour	1.4	2.6
			Suction tip, traction, gland	0.5	26.3
			Cupped forceps, grasping, tumour	0.3	5.3

Incidence reflects the proportional frequency of triplet appearance. Frames represent the proportion of frames in which a given triplet was present, averaged across all analysed videos

**Fig. 2:**
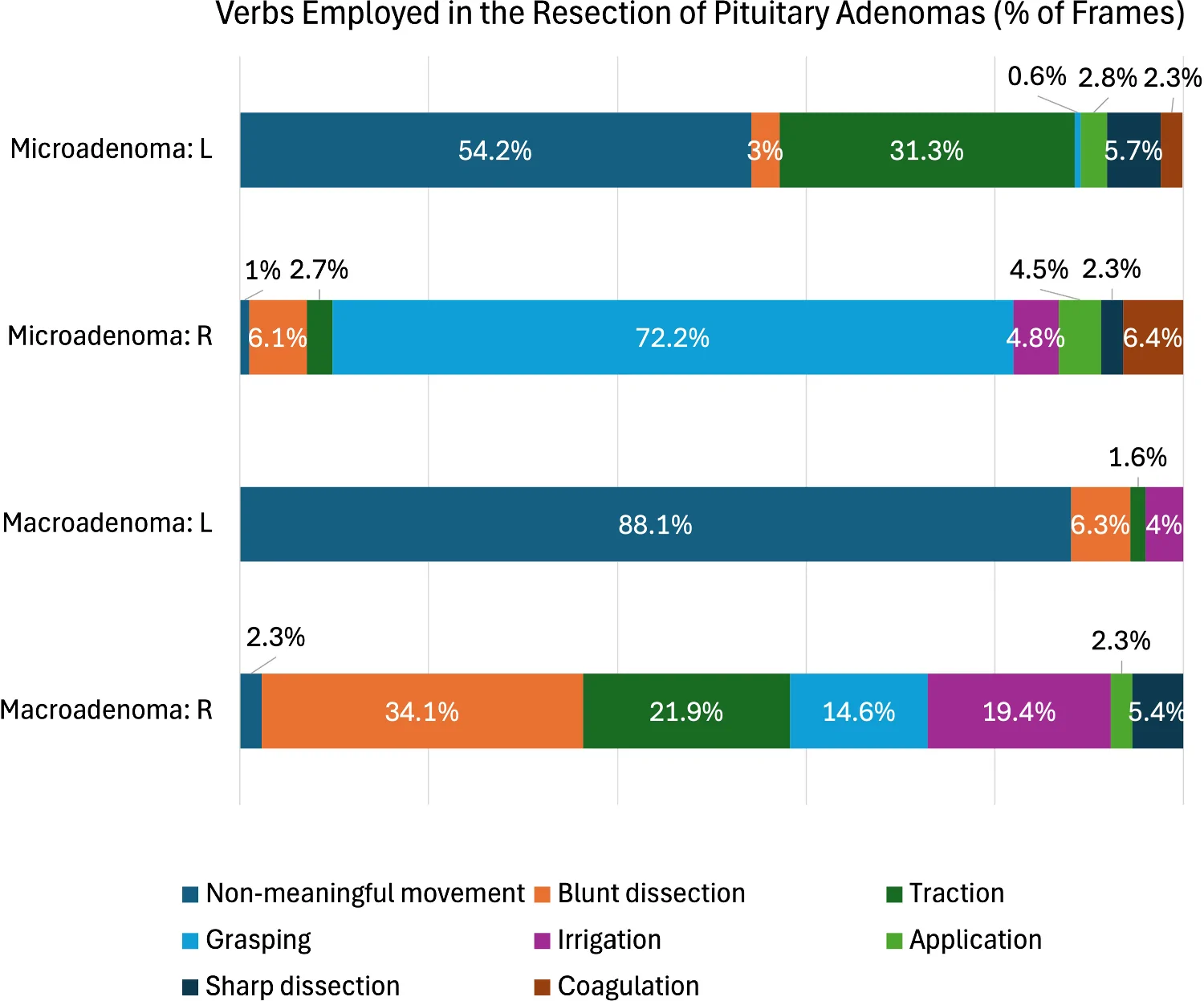
Frame proportions of verbs across pituitary adenoma resections, split according to hand of use

Considering the annotated verbs (Fig. 2), in macroadenoma resections, the right hand’s most common verb was *blunt dissection* (34% of frames), followed by *traction* (21.9%), *irrigation* (19.4%), and *grasping* (14.6%). In the left hand, *non-meaningful movement* was markedly dominant (50.0% incidence, 88.1% of frames). For microadenomas, the right hand’s distribution was markedly different from macroadenomas, with *grasping* accounting for 72% of frames. Similar to macroadenomas, the left hand’s most prevalent verb was *non-meaningful movement* (46.7% incidence, 54.2% of frames). *Traction* (33.3%) occupied a significant 31.3% of frames, contrasting with its much lower frame proportion in macroadenomas.

## Discussion

### Principal findings

We describe a multi-centric, multi-disciplinary, consensus-based approach to developing an annotation ontology for the action triplets involved in the tumour resection step of endoscopic transsphenoidal pituitary surgery. Eighteen surgical videos were annotated to develop a comprehensive and interpretable framework; considering both clinical and data science perspectives. To our knowledge, this is the first study to transparently detail the ontology generation process for surgical actions, via expert-panel review. The defined actions may support objective and automated assessments of surgical performance and procedural complexity, with implications for training and patient outcomes [6], [25–27].

Despite a limited dataset for exploratory comparison across tumour types, the ontology revealed novel associations between surgeon handedness and actions, now scalable using AI methods. In both macro- and microadenoma resections, the left hand (typically controlling suction) predominantly performed *non-meaningful movements* (macroadenoma: 88%, microadenoma 55%), while right-hand instruments were more likely to engage in intentional actions (*Non-meaningful movement*; 1% macro and 2% microadenomas). This may reflect the use of the left-hand suction for triangulation and visualization. For macroadenomas, common right-hand actions were *blunt dissection* and *traction*; for microadenomas, *grasping* dominated (70% of actions). These differences align with anatomical variations, highlighting the ontology’s sensitivity to surgical nuance.

Although the present dataset was not designed to formally compare surgeon performance, several elements of the action ontology show promise as interpretable markers of technical skill. In particular, the high proportion of “non-meaningful movement” and “instrument not in frame” verbs reflects periods in which the instrument is either idle or temporarily lost from the endoscopic field. These behaviours may be associated with lower technical economy and longer task duration. As such, action-level metrics based on the frequency, duration, or temporal clustering of these verbs may serve as objective surrogates for economy of motion, and procedural fluency. Future studies will formally test whether such metrics discriminate skill level or correlate with clinical outcomes.

Inter-rater consistency is crucial for AI training. If experts do not agree on annotations, the application of AI using that ontology is perilous. With a structured annotation guide, even a less experienced annotator achieved “substantial” to “near-perfect agreement” with a more experienced annotator (Cohen’s kappa: 72.7 overall; 75.7 right hand; 69.7 left). This suggests a well-structured annotation guide enhances reproducibility, even among raters with differing expertise, which may reduce the reliance on domain experts for routine labelling tasks. Notably, the higher agreement for the right-hand instrument may reflect its more distinct and intentional movements, whereas the left-hand instrument, often used for suction and traction of tumour tissue, involves more subtle actions that may be harder to categorize consistently. This supports the need for standardized annotation protocols, particularly in complex surgical environments where interpretations can be subjective. Overall, the high inter-rater reliability indicates that the current ontology is well prepared for deployment in AI model training.

Annotation challenges included narrow surgical corridors, causing frequent instrument exits from frame, increasing annotation density [28]. Also, the ring curette frequently switched between the *blunt dissection* and *traction* to mobilize tumour tissue, increasing the annotation density. Furthermore, as is the case for surgical gestures (sub-components of actions), the subjective and often smooth transition between actions makes it challenging to define the temporal boundaries, hindering labelling consistency [29]. Finally, intra-sellar anatomical targets were hard to differentiate due to visual similarity and frequent instrument obstructions at depth, affecting left- hand agreement despite framework iterations.

### Comparison to literature

Operative video analysis beyond the granularity of surgical steps has thus far been limited in the surgical literature. Indeed, the recognition of action triplets has only been described by Nwoye et al. in the context of laparoscopic cholecystectomies, through the CholecT50 dataset [9, 18, 19]. Beyond this, action detection has been restricted to sub-components of the action triplets as whole (instruments, targets, verbs) or verbs (i.e. surgical gestures) in laparoscopic or robotic-assisted procedures. Wagner et al. described action classification using the common verbs used in surgery, including *cut, grasp, hold, and clip*, without instrument or anatomical annotations [18–20]. In robotic surgery, work has focused on classifying gestures (sub-components of actions) through the integration of kinematic data and video data, with mixed results [30]. Ma et al. described the development of a classification system of surgical gestures in robotic hilar dissection, and in their analysis recognized differences between novice and experts [29]. Similarly, in gynaecological surgery, authors [31–33] have approached the challenge of action classification, yet only considering verbs (e.g. *cutting*). Also, the ESAD dataset, released as part of the SARA-ESAD challenge, considers action detection for robotic laparoscopic prostatectomies yet classifies actions based on still images, using only verbs and targets (e.g. “SuckingBlood”) [34].

Beyond the minimal published literature on action recognition as triplet entities, even less work has focused on how annotation frameworks are developed in the first place. This may be an important oversight, considering the importance of label quality on model performance [23, 35]. In a surgical context, the deployment of low-performance models, which may carry inaccuracies, poor generalisability, or annotator biases, has implications for patient safety [24, 36, 37]. Indeed, it has been proposed that prior to large-scale onboarding of data annotators, teams—consisting of AI practitioners and clinicians—should conduct pilot trials, iterating both their labelling frameworks and annotator guidelines, promoting and validating high-quality data labels [24].

This study presents the first annotation ontology for action triplets in neurosurgery, developed through a systematic, consensus-driven approach. By emphasizing universality, clinical relevance, and machine readability, this framework establishes a structured methodology that may serve as a foundation for data-driven improvements in pituitary surgery.

### Strengths and limitations

A key strength of this study is its multi-disciplinary, multi-centric approach, integrating expertise from neurosurgeons and data scientists across two high-volume pituitary centres. This collaborative framework enhances the clinical relevance and machine readability of the ontology, ensuring its comprehensive applicability across disciplines. Additionally, this study prioritizes reproducibility—a critical challenge in AI-driven surgical analysis, where inconsistent labelling can undermine model performance and generalizability. Standardized annotation protocols, paired with a structured user guide, were implemented to mitigate variability, an issue frequently cited in computer vision and biomedical AI research [5]. By emphasizing consistency and interpretability, this work contributes to the growing need for robust, standardized datasets in surgical AI.

While the proposed framework aims to capture the universal spectrum of action triplets in endoscopic pituitary adenoma resection, its generalizability is constrained by the subset of reviewed cases. Surgical techniques vary by institution, surgeon preference, and patient anatomy, meaning certain actions or adaptations—particularly in complex or revision cases—may not be fully represented. Additionally, the dataset is derived from two high-volume pituitary centres, which, while ensuring expertise-driven annotation, may not reflect broader global variations in surgical practice or instrumentation. Expanding the dataset to include a wider range of institutions and training paradigms will be necessary for broader applicability. Furthermore, given the limited number of fully annotated cases, estimates of action distribution and inter-rater reliability should be interpreted as preliminary indicators of feasibility rather than stable quantitative metrics. Accordingly, this work should be viewed as an adaptation of established action-level annotation methodologies to pituitary surgery, providing a foundational framework whose robustness will require validation in larger and more diverse datasets.

Next, although the present framework demonstrates good inter-rater reliability, the scalability of purely manual triplet-level annotation remains a practical limitation. Annotating a single tumour resection step requires several hours of expert time, which restricts dataset growth and limits the feasibility of larger-scale studies. Overcoming this bottleneck will likely require hybrid workflows in which a small manually curated “gold standard” dataset is used to train AI models that then pre-label new videos, with humans acting as verifiers rather than primary annotators. Examples of useful models which could offset at least part of the manual labour include those capable of instrument tracking. Nonetheless, by establishing a standardized ontology and annotation guide, the current work provides the structured foundation necessary for such automation-assisted pipelines.

Finally, a notable challenge in action-level annotation is the inherent fluidity of certain actions. For example, traction and blunt dissection often occur sequentially or simultaneously using the same instrument, resulting in visually ambiguous frames where the temporal boundary between actions is subjective. While our consensus-based ontology reduces this ambiguity, complete resolution using video alone is unlikely. Future work may benefit from integrating multimodal data streams—such as robotic kinematics, or other sensors—which can capture biomechanical signatures (e.g. pulling force vs sweeping motion) that are not visible in 2D video. Such data fusion approaches have already shown promise in gesture-level classification in robotic surgery and may enable more objective triplet segmentation at scale [9].

## Conclusion

The present study employed a multi-centric, multi-disciplinary, consensus-based approach to develop the first action-triplet ontology in neurosurgery. Built upon the principles of operative workflow analysis, this ontology provides a robust framework for future surgical video annotation of larger datasets, facilitating the development of sophisticated AI models, capable of shaping surgical training, predicting outcomes, and helping surgeons make the best decisions for their patients.

## References

[1] Johnson MD, Woodburn CJ, Lee Vance M (2003) Quality of life in patients with a pituitary adenoma. Pituitary 6(2):81–87 14703017 10.1023/b:pitu.0000004798.27230.ed

[2] Lonser RR, Nieman L, Oldfield EH (2017) Cushing’s disease: pathobiology, diagnosis, and management. J Neurosurg 126(2):404–417 27104844 10.3171/2016.1.JNS152119

[3] Biller BMK, Grossman AB, Stewart PM, Melmed S, Bertagna X, Bertherat J, Buchfelder M, Colao A, Hermus AR, Hofland LJ, Klibanski A, Lacroix A, Lindsay JR, Newell-Price J, Nieman LK, Petersenn S, Sonino N, Stalla GK, Swearingen B et al (2008) Treatment of adrenocorticotropin-dependent cushing’s syndrome: a consensus statement. J Clin Endocrinol Metab 93(7):2454–2462 18413427 10.1210/jc.2007-2734 PMC3214276

[4] Ciric I, Ragin A, Baumgartner C, Pierce D (1997) Complications of transsphenoidal surgery: results of a national survey, review of the literature, and personal experience. Neurosurgery 40(2):225–237 9007854 10.1097/00006123-199702000-00001

[5] Meireles OR, Rosman G, Altieri MS, Carin L, Hager G, Madani A, Padoy N, Pugh CM, Sylla P, Ward TM, Hashimoto DA, Ban Y, Filicori F, Mascagni P, Mellinger J, Schlacta C, Speidel S, Juergens T, Garcia-Kilroy P (2021) SAGES consensus recommendations on an annotation framework for surgical video. Surg Endosc 35(9):4918–4929 34231065 10.1007/s00464-021-08578-9

[6] Lalys F, Jannin P (2014) Surgical process modelling: a review. Int J Comput Assist Radiol Surg 9(3):495–511 24014322 10.1007/s11548-013-0940-5

[7] Katić D, Julliard C, Wekerle AL, Kenngott H, Müller-Stich BP, Dillmann R, Speidel S, Jannin P, Gibaud B (2015) LapOntoSPM: an ontology for laparoscopic surgeries and its application to surgical phase recognition. Int J Comput Assist Radiol Surg 10(9):1427–1434 26062794 10.1007/s11548-015-1222-1

[8] Neumuth D, Loebe F, Herre H, Neumuth T (2011) Modeling surgical processes: a four-level translational approach. Artif Intell Med 51(3):147–161 21227665 10.1016/j.artmed.2010.12.003

[9] Nwoye CI, Yu T, Gonzalez C, Seeliger B, Mascagni P, Mutter D, Marescaux J, Padoy N (2022) Rendezvous: attention mechanisms for the recognition of surgical action triplets in endoscopic videos. Med Image Anal 78:102433 35398658 10.1016/j.media.2022.102433

[10] Das A, Khan DZ, Williams SC, Hanrahan JG, Borg A, Dorward NL, Bano S, Marcus HJ, Stoyanov D (2023) A multi-task network for anatomy identification in endoscopic pituitary surgery. Lecture notes in computer science (including subseries Lecture Notes in Artificial Intelligence and Lecture Notes in Bioinformatics). 14228 LNCS:472–82

[11] Das A, Khan DZ, Hanrahan JG, Marcus HJ, Stoyanov D (2023) Automatic generation of operation notes in endoscopic pituitary surgery videos using workflow recognition. Intell-Based Med 8:100107 38523618 10.1016/j.ibmed.2023.100107 PMC10958393

[12] Das A, Bano S, Vasconcelos F, Khan DZ, Marcus HJ, Stoyanov D (2022) Reducing prediction volatility in the surgical workflow recognition of endoscopic pituitary surgery. Int J Comput Assist Radiol Surg 17(8):1445–1452 35362848 10.1007/s11548-022-02599-y PMC9307536

[13] Garrow CR, Kowalewski KF, Li L, Wagner M, Schmidt MW, Engelhardt S, Hashimoto DA, Kenngott HG, Bodenstedt S, Speidel S, Müller-Stich BP, Nickel F (2021) Machine learning for surgical phase recognition: a systematic review. Ann Surg 273(4):684–693 33201088 10.1097/SLA.0000000000004425

[14] Hashimoto DA, Rosman G, Rus D, Meireles OR (2018) Artificial intelligence in surgery: promises and perils. Ann Surg 268(1):70–76 29389679 10.1097/SLA.0000000000002693 PMC5995666

[15] Marcus HJ, Khan DZ, Borg A, Buchfelder M, Cetas JS, Collins JW, Dorward NL, Fleseriu M, Gurnell M, Javadpour M, Jones PS, Koh CH, Layard Horsfall H, Mamelak AN, Mortini P, Muirhead W, Oyesiku NM, Schwartz TH, Sinha S et al (2021) Pituitary society expert Delphi consensus: operative workflow in endoscopic transsphenoidal pituitary adenoma resection. Pituitary 24(6):839–853 34231079 10.1007/s11102-021-01162-3 PMC8259776

[16] Khan DZ, Koh CH, Das A, Valetopolou A, Hanrahan JG, Horsfall HL, Baldeweg SE, Bano S, Borg A, Dorward NL, Olukoya O, Stoyanov D, Marcus HJ (2024) Video-based performance analysis in pituitary surgery—part 1: surgical outcomes. World Neurosurg 190:e787–e796 39122112 10.1016/j.wneu.2024.07.218

[17] Sánchez-González P, Cano AM, Oropesa I, Sánchez-Margallo FM, Pozo FD, Lamata P, Gómez EJ (2011) Laparoscopic video analysis for training and image-guided surgery. Minim Invasive Ther Allied Technol 20(6):311–320 21247251 10.3109/13645706.2010.541921

[18] Wagner M, Müller-Stich BP, Kisilenko A, Tran D, Heger P, Mündermann L, Lubotsky DM, Müller B, Davitashvili T, Capek M, Reinke A, Reid C, Yu T, Vardazaryan A, Nwoye CI, Padoy N, Liu X, Lee EJ, Disch C et al (2023) Comparative validation of machine learning algorithms for surgical workflow and skill analysis with the HeiChole benchmark. Med Image Anal 86:102770 36889206 10.1016/j.media.2023.102770

[19] Nwoye CI, Alapatt D, Yu T, Vardazaryan A, Xia F, Zhao Z, Xia T, Jia F, Yang Y, Wang H, Yu D (2023) CholecTriplet2021: a benchmark challenge for surgical action triplet recognition. Med Image Anal 86:102803 37004378 10.1016/j.media.2023.102803

[20] Twinanda AP, Shehata S, Mutter D, Marescaux J, De Mathelin M, Padoy N (2017) EndoNet: a deep architecture for recognition tasks on laparoscopic videos. IEEE Trans Med Imaging 36(1):86–97 27455522 10.1109/TMI.2016.2593957

[21] TOUCHSURGERY [Internet]. 2025. https://www.touchsurgery.com/

[22] ENCORD [Internet]. 2025. Encord.com

[23] Rädsch T, Reinke A, Weru V, Tizabi MD, Schreck N, Kavur AE, Pekdemir B, Roß T, Kopp-Schneider A, Maier-Hein L (2023) Labelling instructions matter in biomedical image analysis. Nature Mach Intell 5(3):273–283

[24] Freeman B, Hammel N, Phene S, Huang A, Ackermann R, Kanzheleva O, Hutson M, Taggart C, Duong Q, Sayres R (2021) Iterative quality control strategies for expert medical image labeling. Proc AAAI Conf Hum Comput Crowdsour 9:60–71

[25] Dharamsi A, Gray S, Hicks C, Sherbino J, McGowan M, Petrosoniak A (2019) Bougie-assisted cricothyroidotomy: Delphi-derived essential steps for the novice learner. Can J Emerg Med 21(2):283–290 10.1017/cem.2018.386 29952276

[26] Collins JW, Levy J, Stefanidis D, Gallagher A, Coleman M, Cecil T, Ericsson A, Mottrie A, Wiklund P, Ahmed K, Pratschke J, Casali G, Ghazi A, Gomez M, Hung A, Arnold A, Dunning J, Martino M, Vaz C et al (2019) Utilising the Delphi process to develop a proficiency-based progression train-the-trainer course for robotic surgery training. Eur Urol 75(5):775–785 30665812 10.1016/j.eururo.2018.12.044

[27] Lecuyer G, Ragot M, Martin N, Launay L, Jannin P (2020) Assisted phase and step annotation for surgical videos. Int J Comput Assist Radiol Surg 15(4):673–680 32040704 10.1007/s11548-019-02108-8

[28] Marcus HJ, Cundy TP, Hughes-Hallett A, Yang GZ, Darzi A, Nandi D (2014) Endoscopic and keyhole endoscope-assisted neurosurgical approaches: a qualitative survey on technical challenges and technological solutions. Br J Neurosurg 28(5):606 24533591 10.3109/02688697.2014.887654 PMC4032589

[29] Ma R, Vanstrum EB, Nguyen JH, Chen A, Chen J, Hung AJ (2021) A novel dissection gesture classification to characterize robotic dissection technique for renal hilar dissection. J Urol 205(1):271–275 33095096 10.1097/JU.0000000000001328

[30] Van Amsterdam B, Clarkson MJ, Stoyanov D (2021) Gesture recognition in robotic surgery: a review. IEEE Trans Biomed Eng 68(6):2021–2035 33497324 10.1109/TBME.2021.3054828

[31] Khatibi T, Dezyani P (2020) Proposing novel methods for gynecologic surgical action recognition on laparoscopic videos. Multimed Tools Appl 79(41–42):30111–30133

[32] Kletz S, Schoeffmann K, Munzer B, Primus MJ, Husslein H (2017) Surgical action retrieval for assisting video review of laparoscopic skills. In: MultiEdTech 2017 - Proceedings of the 2017 ACM workshop on multimedia-based educational and knowledge technologies for personalized and social online training, co-located with MM 2017.11–9

[33] Petscharnig S, Schoffmann K, Benois-Pineau J, Chaabouni S, Keckstein J (2018) Early and late fusion of temporal information for classification of surgical actions in laparoscopic gynecology. In: Proceedings - IEEE symposium on computer-based medical systems.2018:369–74

[34] Bawa VS, Singh G, KapingA F, Skarga-Bandurova I, Oleari E, Leporini A, Landolfo C, Zhao P, Xiang X, Luo G, Wang K, Li L, Wang B, Zhao S, Li L, Stabile A, Setti F, Muradore R, Cuzzolin F (2021) The SARAS endoscopic surgeon action detection (ESAD) dataset: Challenges and methods. http://arxiv.org/abs/2104.03178

[35] Hsu J, Phene S, Mitani A, Luo J, Hammel N, Krause J, Sayres R (2020) Improving medical annotation quality to decrease labeling burden using stratified noisy cross-validation. http://arxiv.org/abs/2009.10858

[36] Challen R, Denny J, Pitt M, Gompels L, Edwards T, Tsaneva-Atanasova K (2019) Artificial intelligence, bias and clinical safety. BMJ Qual Saf 28(3):231–237 30636200 10.1136/bmjqs-2018-008370 PMC6560460

[37] Zou J, Schiebinger L (2018) AI can be sexist and racist: it’s time to make it fair. Nature 559(7714):324–326 30018439 10.1038/d41586-018-05707-8

